# Performance Analysis of Wireless Information Surveillance in Machine-Type Communication at Finite Blocklength Regime

**DOI:** 10.3390/s19133031

**Published:** 2019-07-09

**Authors:** Ruonan Dong, Baogang Li, Binyang Yan

**Affiliations:** Department of Electronic and Communication Engineering, North China Electric Power University, No. 619, Yong Hua Street, Baoding 071003, China

**Keywords:** wireless information surveillance, proactive eavesdropping, finite blocklength, channel coding rate, IoT, machine-type communication

## Abstract

The Internet of Things (IoT) will feature pervasive sensing and control capabilities via the massive deployment of machine-type communication devices in order to greatly improve daily life. However, machine-type communications can be illegally used (e.g., by criminals or terrorists) which is difficult to monitor, and thus presents new security challenges. The information exchanged in machine-type communications is usually transmitted in short packets. Thus, this paper investigates a legitimate surveillance system via proactive eavesdropping at finite blocklength regime. Under the finite blocklength regime, we analyze the channel coding rate of the eavesdropping link and the suspicious link. We find that the legitimate monitor can still eavesdrop the information sent by the suspicious transmitter as the blocklength decreases, even when the eavesdropping is failed under the Shannon capacity regime. Moreover, we define a metric called the effective eavesdropping rate and study the monotonicity. From the analysis of monotonicity, the existence of a maximum effective eavesdropping rate for a moderate or even high signal-to-noise (SNR) is verified. Finally, numerical results are provided and discussed. In the simulation, we also find that the maximum effective eavesdropping rate slowly increases with the blocklength.

## 1. Introduction

The vision of the Internet of Things (IoT) promises to bring wireless connectivity to anything ranging from tiny static sensors to vehicles and unmanned aerial vehicles (UAVs) [1,2,3]. Meanwhile, short packets are the typical form of traffic generated by sensors and exchanged in machine-type communications [4]. In these scenarios, the Shannon capacity, which assumes the infinite blocklength, is no longer achievable. In comparison to the Shannon capacity regime, reference [5] developed a pioneering framework and identified a tight bound of the channel coding rate at the finite blocklength regime, which presents many new research opportunities with a wide range of applications.

The IoT can offer many benefits for daily life; however, machine-type communications, such as vehicle to vehicle communication and UAV communication among others, can be illegally used (e.g., by criminals or terrorists), which is difficult to monitor, thus presenting new challenges with respect to public security [6]. Thus, legitimate eavesdropping by legitimate parties should be necessary to effectively discover and prevent the information transmitted between the suspicious users. Further, proactive eavesdropping has recently attracted much interest in research as an approach to improve eavesdropping performance.

### 1.1. Related Works

Conventional wireless security studies generally assume wireless communication is rightful, i.e., the eavesdropper is treated as an adversary, and aim to preserve their confidentiality and prevent malicious eavesdropping [7,8]. In the presence of a malicious eavesdropper, the network of point-to-point [7], relaying [8,9], multi-user [10,11], and cognitive radio [12] were investigated. In contrast, legitimate eavesdropping or wireless information surveillance is a paradigm shift of wireless security, where the monitor is regarded as a legitimate eavesdropper.

In general, there are two approaches for wireless information surveillance, including passive eavesdropping and proactive eavesdropping. With passive eavesdropping, the legitimate monitor only listens to the wireless channels of the suspicious users. This approach can’t change the eavesdropping performance. However, proactive eavesdropping can generally improve the eavesdropping performance via jamming or relaying. Note that there is not much research on the legitimate proactive eavesdropping in the literature, where the legitimate monitor eavesdrops a single suspicious link [13,14,15,16,17,18,19,20,21], multiple suspicious links [22,23], or a suspicious relaying link [24,25,26]. A legitimate surveillance scenario where a legitimate monitor aimed to eavesdrop a point-to-point suspicious communication link via jamming [13] and cognitive jamming [14,15] was investigated, and the eavesdropping rate at the legitimate monitor was studied. In [16], the author studied the legitimate surveillance system consisting of two legitimate monitors. In [17,18], the legitimate monitor was equipped with multiple antennae and acted as a fake relay to eavesdrop the suspicious transmitter–receiver pair. In [19,20,21], the author studied a new spoofing approach to change the communicated information of the suspicious link. The work in [22] investigated the wireless surveillance of multiple suspicious links, and maximized weighted sum eavesdropping rate of multiple suspicious links. The work in [23] studied the wireless surveillance of multiple suspicious communication links and proposed a cooperative eavesdropping scheme. The eavesdropping rate [24], the eavesdropping mode [25], and the eavesdropping non-outage probability [26] were studied where the legitimate monitor aims to eavesdrop a suspicious relaying communication link.

### 1.2. Contributions and Organizations

As a common point, all the above studies are under the Shannon capacity regime, where the length of the block is assumed to be infinite. The Shannon capacity is not achievable when the information transmitted in short packets. To our best knowledge, there is no research on the legitimate proactive eavesdropping under the finite blocklength regime. Therefore, this paper analyzes the performance of a legitimate surveillance system via proactive eavesdropping at the finite blocklength regime. In the system, there is a suspicious transmitter-receiver pair, which may be two stationary UAVs etc, and a legitimate monitor. The legitimate monitor operates in a full-duplex mode with simultaneous information reception and relaying. The main contributions are summarized as follows.

In this paper, under the finite blocklength regime, we analyze the channel coding rate of the eavesdropping link and the suspicious link. Meanwhile, we find that the legitimate monitor can still eavesdrop the information sent by the suspicious transmitter as the blocklength decreases, even when the eavesdropping is failed under the Shannon capacity regime. Moreover, we define a metric called the effective eavesdropping rate and analyze the monotonicity. From the analysis of monotonicity, the existence of a maximum effective eavesdropping rate for moderate or even high signal-to-noise (SNR) is verified. Finally, numerical results are provided and discussed. In the simulation, we also find that the maximum effective eavesdropping rate slowly increases with the blocklength, and the increment is almost negligible when the blocklength reaches a relatively large value.

The rest of this paper is organized as follows. The system model and assumptions are described in Section 2. Section 3 analyzes the performance of the legitimate surveillance system at finite blocklength. Numerical results are presented in Section 4. Finally, the paper is concluded in Section 5.

## 2. System Model and Assumptions

**Figure 1:**
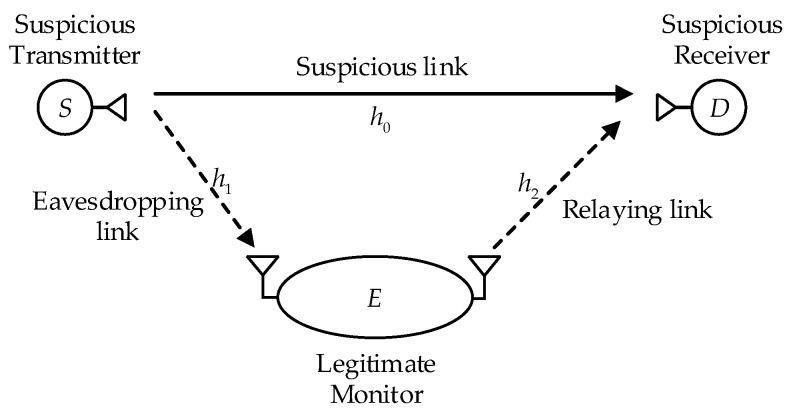
System model of the considered legitimate surveillance system.

As shown in Figure 1, we consider a legitimate surveillance system consisting of a suspicious transmitter-receiver pair (i.e., *S*-*D*) and a full-duplex legitimate monitor *E*. *S* transmits information to *D* during *n* channel uses, in this way, we consider that each block spans over *n* channel uses. We assume that both *S* and *D* are unaware of the presence of *E* and the decode-and-forward (DF) relaying is adopted by *E*. If *E* decodes the block received from *S* successfully, it forwards the block to *D*, which aims to enhance eavesdropping the suspicious link. *S* and *D* are each equipped with a single antenna, and *E* is equipped with two antennae, one for eavesdropping (receiving) and the other for relaying (transmitting). *S* can adaptively adjust its transmission rate. The self-interference from the relaying antenna to the eavesdropping antenna at the legitimate monitor is assumed to be perfectly cancelled by using advanced analog and digital self-interference cancellation methods [13]. DF can be assumed here as in [8,27]. In addition, *E* can act as a fake relay and thus obtain the channel state information and the symbol format of the suspicious link, and synchronize with *S* and *D* [19,20].

We consider a Rayleigh quasi-static block-fading channel [28], where fading process is considered to be constant over the transmission of a block and independently and identically distributed from block to block. Let $h_{0}$, $h_{1}$ and $h_{2}$ denote channel coefficients from the suspicious transmitter to the suspicious receiver, from the suspicious transmitter to the eavesdropping antenna of the legitimate monitor, and from the relaying antenna of the legitimate monitor to the suspicious receiver, respectively. The corresponding channel gains are defined as $g_{0}={\left|h_{0}\right|}^{2}$, $g_{1}={\left|h_{1}\right|}^{2}$ and $g_{2}={\left|h_{2}\right|}^{2}$. In addition, we assume that *E* perfectly knows the channel state information of all links, which can be obtained by utilizing the methods given in the literature [14,17,19,20].

### Channel Coding Rate for Finite Blocklength

For a given decoding error probability ε, the channel coding rate *R* (in bits per channel use) with blocklength *n* is [28,29]

(1) $$R=C-\sqrt{\left(1-1/{\left(1+\gamma \right)}^{2}\right)/n}\cdot Q^{-1}\left(\varepsilon \right)\log_{2}e$$

where $Q^{-1}\left(.\right)$ is the inverse *Q*-function and as usual the *Q*-function is given by $Q\left(x\right)=\int_{x}^{\infty }\frac{1}{\sqrt{2\pi }}e^{-t^{2}/2}dt$. In addition, $C=\log_{2}(1+\gamma )$ is Shannon capacity function of the SNR γ. Note that Equation (1) is a very tight approximation when n≥100, i.e., the difference from the exact value can be neglected [28,29]. Thus, we consider n≥100 in this paper and use equal sign in Equation (1). Based on the above results, *R* can be transformed into

(2) $$R=C-\sqrt{\left(1-2^{-2C}\right)/n}\cdot Q^{-1}\left(\varepsilon \right)\log_{2}e$$

Equivalently, for a given channel coding rate *R*, the decoding error probability ε can be given by

(3) $$\varepsilon =Q\left(\frac{C-R}{\sqrt{\left(1-1/{\left(1+\gamma \right)}^{2}\right)/n}\cdot \log_{2}e}\right)=Q\left(\frac{C-R}{\sqrt{\left(1-2^{-2C}\right)/n}\cdot \log_{2}e}\right)$$

## 3. Performance at Finite Blocklength

In this section, under the finite blocklength regime, we first analyze the performance of the legitimate surveillance system in terms of the channel coding rate of the eavesdropping link and the suspicious link in comparison with the Shannon capacity regime. Afterwards, we define a metric called the effective eavesdropping rate and analyze the monotonicity. From the analysis of monotonicity, the existence of a maximum effective eavesdropping rate for moderate or even high SNR is also verified.

### 3.1. Analysis of Channel Coding Rate

According to Equation (2), the channel coding rate of the eavesdropping link can be obtained as

(4) $$R_{E}=C_{E}-\sqrt{\left(1-2^{-2C_{E}}\right)/n}\cdot Q^{-1}\left(\varepsilon_{E}\right)\log_{2}e$$

where $C_{E}=\log_{2}(1+\gamma_{E})$, $\gamma_{E}=g_{1}P_{1}/\sigma_{E}{}^{2}$ is the SNR at *E*, $P_{1}$ is the transmit power at *S*, $\sigma_{E}{}^{2}$ is the power of noise at *E*, and $\varepsilon_{E}$ is the decoding error probability at *E*. Likewise, the effective channel coding rate of the suspicious link can be obtained as

(5) $$R_{D}=C_{D}-\sqrt{\left(1-2^{-2C_{D}}\right)/n}\cdot Q^{-1}\left(\varepsilon_{D}\right)\log_{2}e$$

where $C_{D}=\log_{2}(1+\gamma_{D})$, $\gamma_{D}=\left(g_{0}P_{1}+g_{2}P_{2}\right)/\sigma_{D}{}^{2}$ is the effective SNR at *D*, $P_{2}$ is the transmit power at *E*, $\sigma_{D}{}^{2}$ is the power of noise at *D*, and $\varepsilon_{D}$ is the decoding error probability at *D*. *E* can act as a fake relay and alter the effective channel of the suspicious link from *S* to *D* [17]. Thus, we use effective channel coding rate, which includes the suspicious link and the relaying link. $\varepsilon_{D}$ results from the error probability of each link and is given by

(6) $$\varepsilon_{D}=\varepsilon_{0}\left[\varepsilon_{E}+\left(1-\varepsilon_{E}\right)\varepsilon_{2}\right]$$

where $\varepsilon_{0}$ and $\varepsilon_{2}$ are the decoding error probabilities of the suspicious link and the relaying link, respectively.

Since $(1-\varepsilon_{E})(1-\varepsilon_{2})\geq 0$, it is straightforward to know that $\varepsilon_{E}+\varepsilon_{2}-\varepsilon_{E}\varepsilon_{2}\leq 1$. Thus, we immediately have $\varepsilon_{D}\leq \varepsilon_{0}$. Besides we consider that $\varepsilon_{E}\geq \varepsilon_{2}$, in this way, we have $\varepsilon_{D}=\varepsilon_{0}\varepsilon_{E}(1-\varepsilon_{2})+\varepsilon_{0}\varepsilon_{2}\leq \varepsilon_{0}\varepsilon_{E}+\varepsilon_{0}\varepsilon_{2}\leq 2\varepsilon_{0}\varepsilon_{E}$. In summary, we can obtain as follows

(7) $$\varepsilon_{D}\leq \varepsilon_{0}\cdot \min\{2\varepsilon_{E},1\}$$

It can be known that Q(x)<0.5 when x>0. So according to Equation (3), ε<0.5. In this way, we immediately have $\varepsilon_{E}<0.5$. Thus, we can derive $\varepsilon_{D}<\varepsilon_{E}$ from Equation (7).

When $\varepsilon_{E}<\varepsilon_{2}$, we can obtain $\varepsilon_{D}<\varepsilon_{2}$. But, we consider $\varepsilon_{E}\geq \varepsilon_{2}$ is more reasonable. The reasons mainly include the following: $\varepsilon_{2}$ decreases as the transmission rate of *E* decreases; $\varepsilon_{2}$ decreases as the transmit power of *E* increases; meanwhile, as the transmit power of *E* increases, $\varepsilon_{E}$ increases. Overall, $\varepsilon_{2}$ can be controlled at a very small value by reducing the transmission rate of *E* or increasing the transmit power of *E*.

In general, under the Shannon capacity regime, the Shannon capacity of the eavesdropping link is $C_{E}$, accordingly, the effective Shannon capacity of the suspicious link is $C_{D}$, as in [17]. Next, we give the following proposition.

**Proposition** **1:** *$R_{E}>R_{D}$ when $C_{E}>C_{D}$, i.e., under the finite blocklength regime, E can eavesdrop the information sent by S the same as the condition under the Shannon capacity regime.*

**Figure 2:**
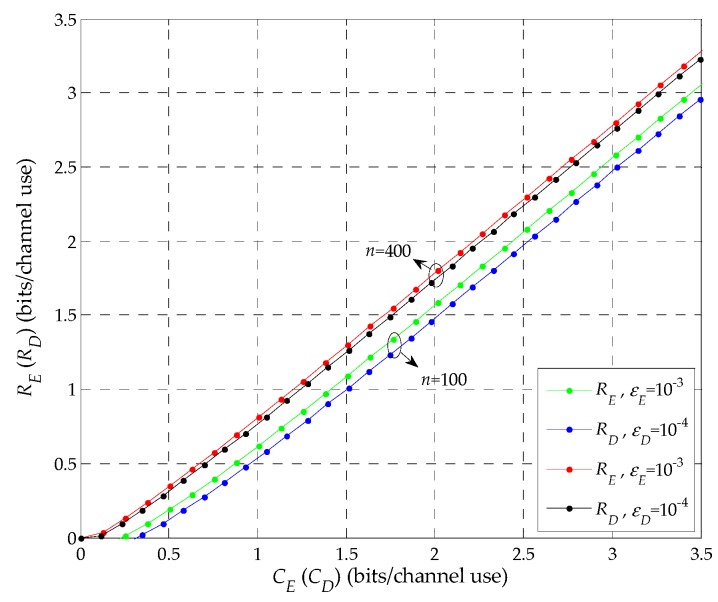
$R_{E}$ vs $C_{E}$ and $R_{D}$ vs $C_{D}$.

**Proof:**  See detailed proof of Proposition 1 in Appendix A. The corresponding simulation is shown in Figure 2. □

Next, we give the following proposition, which is different from the results under the Shannon capacity regime where the legitimate monitor can eavesdrop the information sent by the suspicious transmitter only when $C_{E}\geq C_{D}$.

**Proposition** **2:** *E can still eavesdrop the information sent by S as n decreases even though in some conditions of*$C_{E}<C_{D}$*, i.e., when n decreases,*$R_{E}\geq R_{D}$*can still be achieved even in some conditions of*$C_{E}<C_{D}$.

**Figure 3:**
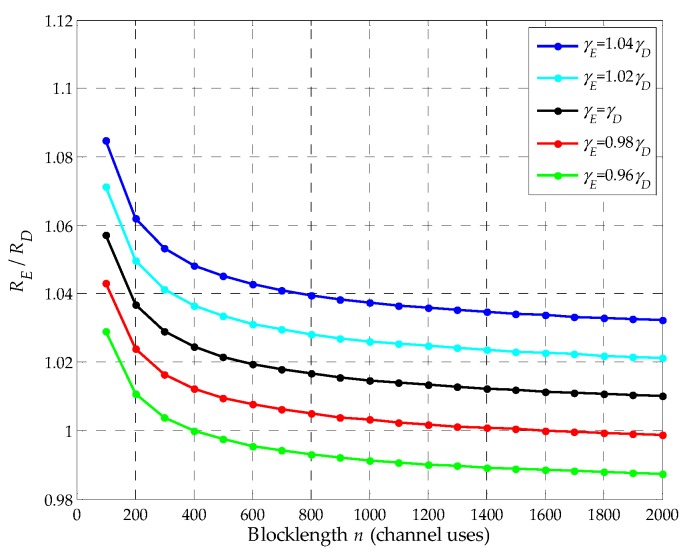
The ratio of $R_{E}$ and $R_{D}$ as a function of the blocklength *n*.

**Proof:**  Based on Equation (A1), it is known that $R_{E}-R_{D}>0$ when $C_{E}=C_{D}$. Further, according to Equation (A1), the value of $R_{E}-R_{D}$ decreases with *n* because *n* is in the denominator. Therefore, the value of $R_{E}-R_{D}$ increases as *n* decreases. In this way, in some conditions of $C_{E}<C_{D}$, $R_{E}\geq R_{D}$ can still be achieved as *n* decreases, which is investigated by simulation in Figure 3. Thus, *E* can still eavesdrop the information sent by *S* as *n* decreases even though in some conditions of $C_{E}<C_{D}$.□

### 3.2. Analysis of Effective Eavesdropping Rate

When $R_{E}>R_{D}$, there is always a potential chance, such as increasing the relaying power of the legitimate monitor, to improve the eavesdropping rate by increasing $R_{D}$ until $R_{E}=R_{D}$, which means that $R_{D}$ reaches the optimal value. Then, any more improvement of $R_{D}$ will lead to $R_{E}<R_{D}$, which means the failure of eavesdropping. So, when the suspicious link is eavesdropped with optimal eavesdropping rate, the relation of $R_{E}=R_{D}$ is always realized.

Next, under the finite blocklength regime, we define a metric called effective eavesdropping rate to analyze the system performance. Mathematically, the effective eavesdropping rate is given by

(8) $$R_{eff}=R_{eav}(1-\varepsilon_{E})$$

where $R_{eav}$ is the eavesdropping rate, and $R_{eav}=R_{D}=R_{E}$. According to Equation (3), we can reformulate Equation (8) as a function of $R_{eav}$ as

(9) $$R_{eff}=R_{eav}\left(1-Q\left(\frac{a-R_{eav}}{b}\right)\right)$$

where $a=C_{E}=\log_{2}\left(1+\gamma_{E}\right)$, and $b=\sqrt{\left(1-\frac{1}{{\left(1+\gamma_{E}\right)}^{2}}\right)/n}\cdot \log_{2}e$. Next, we study Equation (9), for which we have the following lemma.

**Lemma** **1:** Under the finite blocklength regime, the effective eavesdropping rate $R_{eff}$ is monotonically increasing over $\left[0,R_{eav}^{*}\right]$ and monotonically decreasing over $\left(R_{eav}^{*},a\right)$ for moderate or even high SNR, where $R_{eav}^{*}$ is the eavesdropping rate that maximizes the effective eavesdropping rate $R_{eff}$.

**Proof:**  See detailed proof of Lemma 1 in Appendix B. □

**Figure 4:**
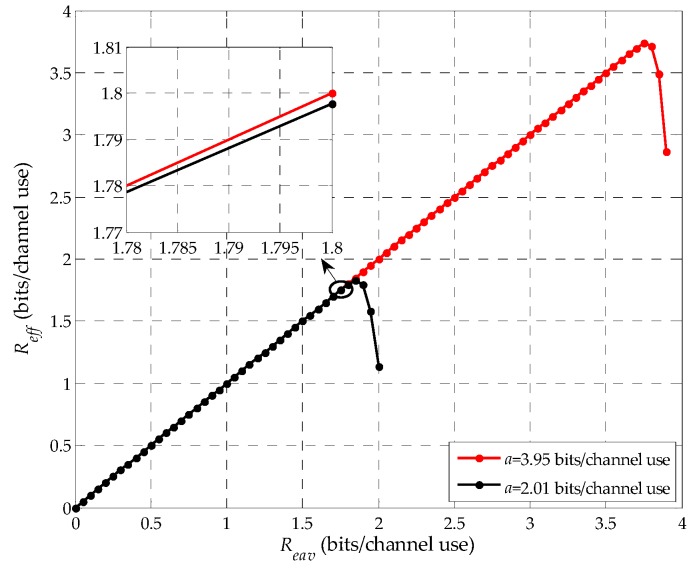
$R_{eff}$ vs $R_{eav}$ given in Equation (9).

Base on the proof of Lemma 1, we prove that there exists a maximum effective eavesdropping rate, $R_{eff}^{*}$, corresponding to $R_{eav}^{*}$. However, unfortunately, the general closed-form for $R_{eav}^{*}$ cannot be derived. Therefore, it is investigated by simulation in Figure 4. Furthermore, we consider the optimal eavesdropping rate $R_{eav}^{opt}=\max\left(R_{eav}^{*},R_{0}\right)$, where $R_{0}$ is the channel coding rate of the suspicious link with no relaying power. Here, we first simply explain it as follows. We consider the eavesdropping rate $R_{0}\leq R_{eav}<a$. First, consider the case when $R_{eav}^{*}\geq R_{0}$. In this case, the legitimate monitor should use a positive relaying power to facilitate the eavesdropping, such that the effective channel coding rate $R_{D}$ of the suspicious link is improved from $R_{0}$ to $R_{eav}^{*}$, thus, we have $R_{eav}^{opt}=R_{eav}^{*}$ and the optimal effective eavesdropping rate $R_{eff}^{opt}=R_{eff}^{*}$. Next, consider $R_{eav}^{*}<R_{0}$. In this case, we have $R_{eav}^{opt}=R_{0}$, which means that no relaying is required for the legitimate monitor to obtain its optimal effective eavesdropping rate.

## 4. Numerical Results

Next, we present numerical results obtained by simulations for the considered legitimate surveillance system. We consider the Rayleigh quasi-static block-fading channel and set the channel coefficients $h_{0}$, $h_{1}$ and $h_{2}$ to be independent circularly symmetric complex Gaussian random variables with mean zero and variance 1. Here, the transmit powers are normalized over the receiver noise powers such that we can set the noise powers at *E* and *D* to be $\sigma_{E}{}^{2}=\sigma_{D}^{2}=1$. Unless otherwise stated, we set the transmit power at *S* as $P_{1}=20$ dB. We assume that the transmit power $P_{2}$ is large enough to facilitate the eavesdropping.

In Figure 2, $R_{E}$ with $C_{E}$ and $R_{D}$ with $C_{D}$ are shown for given blocklength *n* and error probability ε. Here, the transmit power $P_{2}$ is set to be 2 dB. Without loss of generality, *n* is set to be 100 and 400 channel uses, $\varepsilon_{E}$ and $\varepsilon_{D}$ are set to be 10^−3^ and 10^−4^, respectively. As shown in the figure, when $C_{E}\geq C_{D}$, it is clear that $R_{E}>R_{D}$. Meanwhile, we can note that $R_{E}$ increases with $C_{E}$, and that $R_{D}$ also increases with $C_{D}$. For example, when *n* is 400 channel uses, for $C_{E}=C_{D}=1.63$, $R_{E}-R_{D}=0.04$, while for $C_{E}=2.14$ and $C_{D}=2.1$, $R_{E}-R_{D}=0.09$, so $R_{E}-R_{D}>0$ when $C_{E}\geq C_{D}$. Thus, under the finite blocklength regime, *E* can eavesdrop the information sent by *S* the same as the condition under the Shannon capacity regime, which is in line with Proposition 1.

In Figure 3, we plot the ratio of $R_{E}$ and $R_{D}$ with *n* when $\gamma_{E}=1.04\gamma_{D}$, $\gamma_{E}=1.02\gamma_{D}$, $\gamma_{E}=\gamma_{D}$, $\gamma_{E}=0.98\gamma_{D}$ and $\gamma_{E}=0.96\gamma_{D}$, where $\gamma_{E}=0.98\gamma_{D}$ and $\gamma_{E}=0.96\gamma_{D}$ represent some conditions of $C_{E}<C_{D}$. We set $\varepsilon_{E}$ and $\varepsilon_{D}$ to be 10^−3^ and 10^−4^, respectively. As shown in the figure, we can note that when $\gamma_{E}\geq \gamma_{D}$, $R_{E}/R_{D}>1$ and $R_{E}/R_{D}$ decreases with *n*. Meanwhile, in comparison to $\gamma_{E}=\gamma_{D}$, $R_{E}/R_{D}$ can still be larger than or equal to 1 when $\gamma_{E}=0.98\gamma_{D}$ and $\gamma_{E}=0.96\gamma_{D}$ as shown in the figure. For example, when $R_{E}/R_{D}=1$, the blocklengths *n* of the red and green curves are respectively around 1400, 400 channel uses, thus, *n* decreases. So even in some conditions of $C_{E}<C_{D}$, *E* can still eavesdrop the information sent by *S* as *n* decreases, which demonstrates proposition 2.

Figure 4 shows the effective eavesdropping rate $R_{eff}$ with the eavesdropping rate $R_{eav}$ at *E* given in Equation (9). Here, the results are obtained when *a* is 2.01 and 3.95 bits per channel use, thus, we can obtain that $\gamma_{E}$ is 4.81 dB and 11.6 dB, which are supposed to moderate SNRs. Without loss of generality, we set *n* to be 400 channel uses. As shown in the figure, we can note that $R_{eff}$ is first monotonically increasing and then monotonically decreasing and there is a maximum value of the eavesdropping rate, $R_{eav}^{*}$, which is corresponding to the maximum value of the effective eavesdropping rate, $R_{eff}^{*}$. For example, $R_{eav}^{*}$ is around 3.7 when $\gamma_{E}$ is 11.6 dB. Moreover, we can also note that $R_{eff}$ is larger when $\gamma_{E}$ is 11.6 dB compared with $\gamma_{E}$ is 4.81 dB. Thus, for a given blocklength n, $R_{eff}$ increases with $\gamma_{E}$ for the same $R_{eav}$. So far, the Lemma 1 is demonstrated by simulation.

**Figure 5:**
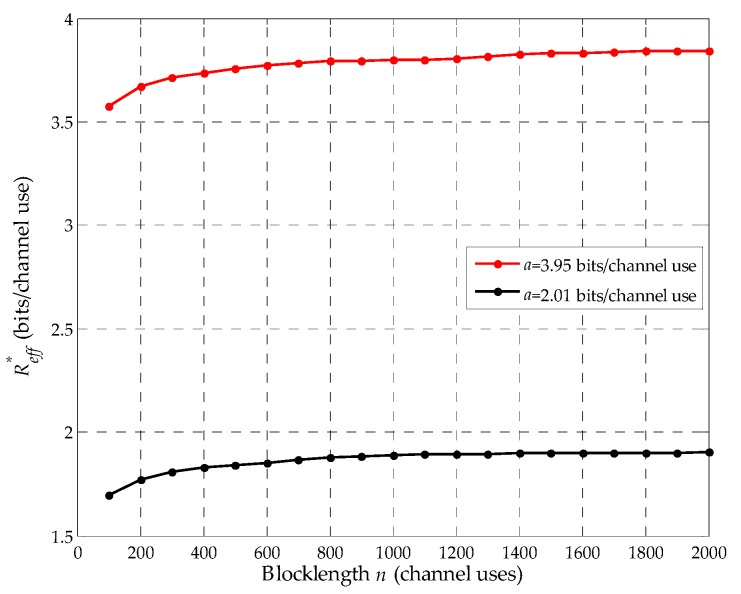
$R_{eff}^{*}$ vs the blocklength *n*.

In Figure 5, we plot the maximum effective eavesdropping rate $R_{eff}^{*}$ with the blocklength *n*. Here, corresponding to Figure 4, the results are obtained when *a* is 2.01 and 3.95 bits per channel use. As show in Figure 5, we can clearly note that $R_{eff}^{*}$ increases with *n*. We can also note that the increments of the curves are almost negligible when *n* reaches a relatively large value. For example, the increment of the red curve is very small in the range of 1500 channel uses to 2000 channel uses. Moreover, it is easy to see that $R_{eff}^{*}$ increases with *a*, thus, $R_{eff}^{*}$ increases with $\gamma_{E}$.

## 5. Conclusions

In this paper, under the finite blocklength regime, we analyze the performance of a legitimate proactive eavesdropping system, which consists of a suspicious transmitter–receiver pair and a legitimate monitor. We consider that the legitimate monitor operates in a full-duplex mode with simultaneous information reception and relaying. Moreover, we analyze the channel coding rate of the eavesdropping link and the suspicious link. We find that the legitimate monitor can still eavesdrop the information sent by the suspicious transmitter as the blocklength decreases, even when the eavesdropping is failed under the Shannon capacity regime. Furthermore, we define a metric called effective eavesdropping rate and analyze the monotonicity. From the analysis of monotonicity, the existence of a maximum effective eavesdropping rate for moderate or even high SNR is verified. Finally, numerical results are provided and discussed. In the simulation, we also find that the maximum effective eavesdropping rate slowly increases with the blocklength, and the increment is almost negligible when the blocklength is relatively large.

## Figures and Tables

**Figure A1:**
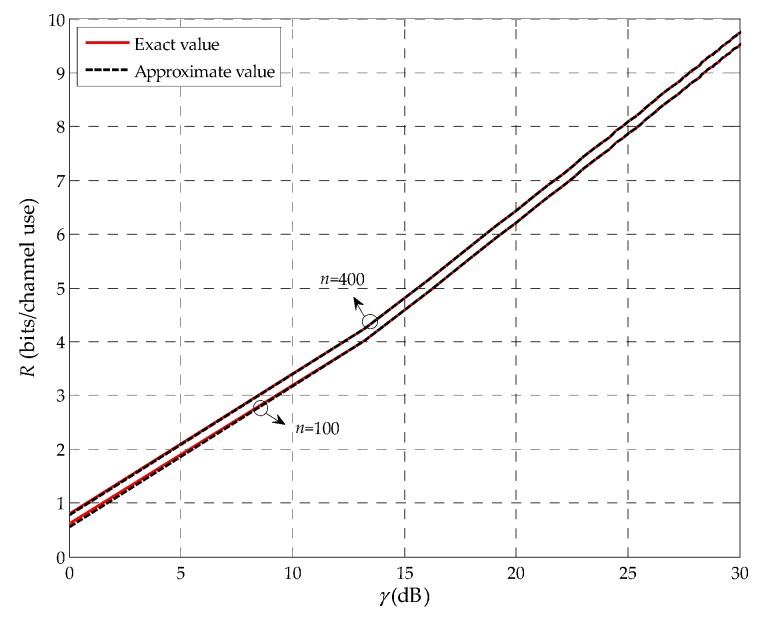
*R* with γ via Equation (1) and Equation (A2).

## Appendix A

**Proof of Proposition 1**  First, when $C_{E}=C_{D}$, we have

(A1) $$\begin{array}{lll} R_{E}-R_{D} & = & C_{E}-\sqrt{\left(1-2^{-2C_{E}}\right)/n}\cdot Q^{-1}\left(\varepsilon_{E}\right)\log_{2}e\\ & & -\left(C_{D}-\sqrt{\left(1-2^{-2C_{D}}\right)/n}\cdot Q^{-1}\left(\varepsilon_{D}\right)\log_{2}e\right)\\ & = & \sqrt{\left(1-2^{-2C_{D}}\right)/n}\cdot Q^{-1}\left(\varepsilon_{D}\right)\log_{2}e\\ & & -\sqrt{\left(1-2^{-2C_{E}}\right)/n}\cdot Q^{-1}\left(\varepsilon_{E}\right)\log_{2}e\\ & = & \sqrt{\left(1-2^{-2C_{D}}\right)/n}\cdot \log_{2}e\cdot \left[Q^{-1}\left(\varepsilon_{D}\right)-Q^{-1}\left(\varepsilon_{E}\right)\right] \end{array}$$

where it can be known that $\sqrt{\left(1-2^{-2C_{D}}\right)/n}\cdot \log_{2}e>0$. We have obtained $\varepsilon_{D}<\varepsilon_{E}$, so we can derive $Q^{-1}\left(\varepsilon_{D}\right)>Q^{-1}\left(\varepsilon_{E}\right)$ by using the fact that $Q^{-1}\left(x\right)$ is the decreasing function of *x*. Thus, we can obtain $R_{E}>R_{D}$ when $C_{E}=C_{D}$. Afterwards, Equation (1) can be approximated as

(A2) $$R=C-\sqrt{1/n}\cdot Q^{-1}\left(\varepsilon \right)\log_{2}e$$

As is shown in the Figure A1, the approximation, i.e. Equation (A2), is very tight for the range of SNR.
According to Equation (A2), it can be known that R increases with C. Thus, $R_{D}$ increases with $C_{D}$, therefore, if $C_{E}>C_{D}$, which means that $C_{D}$ is smaller in comparison with the condition $C_{E}=C_{D}$, $R_{E}$ is definitely larger than $R_{D}$. In conclusion, $R_{E}>R_{D}$ when $C_{E}\geq C_{D}$. □

## Appendix B

**Proof of Lemma 1**  To demonstrate there is the value of $R_{eav}$ that maximizes the effective eavesdropping rate $R_{eff}$, we next examine the monotonicity and concavity of $R_{eff}$ with respect to $R_{eav}$. For this purpose, we derive the first and second derivatives of $R_{eff}$ with respect to $R_{eav}$ respectively. Based on the differentiation of a definite integral in terms of a parameter [30], the first derivative of $R_{eff}$ with respect to $R_{eav}$ is given by

(A3) $$\begin{array}{l} R_{eff}{}^{\prime }\left(R_{eav}\right)=\left(1-Q\left(\frac{a-R_{eav}}{b}\right)\right)+R_{eav}\cdot \left(-\frac{m}{b}\right)\\ =1-Q\left(\frac{a-R_{eav}}{b}\right)-\frac{R_{eav}m}{b} \end{array}$$

where $m=\frac{1}{\sqrt{2\pi }}e^{-\frac{{\left(a-R_{eav}\right)}^{2}}{2b^{2}}}$. Likewise, the second derivative of $R_{eff}$ with respect to $R_{eav}$ is obtained as

(A4) $$\begin{array}{l} R_{eff}{}^{\prime \prime }\left(R_{eav}\right)=-\frac{m}{b}-\left(\frac{m}{b}+\frac{R_{eav}m\left(a-R_{eav}\right)}{b^{3}}\right)\\ =-\frac{2m}{b}-\frac{R_{eav}m\left(a-R_{eav}\right)}{b^{3}} \end{array}$$

We can easily note that a>0 and b>0. In this way, we can immediately obtain that

(A5) $$R_{eff}{}^{\prime }\left(0\right)=1-Q\left(\frac{a}{b}\right)>0$$

which is due to $0<Q\left(\frac{a}{b}\right)<0.5$. Besides, we can also obtain that

(A6) $$R_{eff}{}^{\prime \prime }\left(0\right)=-\frac{2m\left(0\right)}{b}<0$$

which is due to m>0. Moreover, we find that $R_{eff}^{\prime \prime }\left(R_{eav}\right)<0$ within $0\leq R_{eav}<a$. So, ${R^{\prime }}_{eff}\left(R_{eav}\right)$ keeps decreasing in the range of $0\leq R_{eav}<a$. We next confirm that the value of $R_{eff}^{\prime }\left(a\right)$ is larger than zero or smaller than zero. According to Equation (A3), we have

(A7) $$\begin{array}{ll} R_{eff}{}^{\prime }\left(a\right) & =1-Q\left(0\right)-\frac{am\left(a\right)}{b}\\ & =0.5-\frac{a}{b\sqrt{2\pi }}\\ & =0.5-\frac{\log_{2}(1+\gamma_{E})}{\sqrt{\left(1-\frac{1}{{\left(1+\gamma_{E}\right)}^{2}}\right)/n}\cdot \log_{2}e\cdot \sqrt{2\pi }} \end{array}$$

It is easy to know that the value of $R_{eff}^{\prime }\left(a\right)$ decreases as $\gamma_{E}$ increases, and also decreases as *n* increases. In general, the SNR is relatively small when $\gamma_{E}=-5$ dB. Note that Equation (3) is just an approximation when n is large enough [29], e.g. n≥100. By bringing $\gamma_{E}=-5$ dB and n=100 channel uses into Equation (A7), we obtain that $R_{eff}^{\prime }\left(a\right)<0$. So for moderate or even high SNR, $R_{eff}^{\prime }\left(a\right)$ is definitely smaller than zero with a given n. Summarizing, $R_{eff}^{\prime }\left(R_{eav}\right)$ keeps decreasing within $0\leq R_{eav}<a$, meanwhile $R_{eff}^{\prime }\left(0\right)>0$ and $R_{eff}^{\prime }\left(a\right)<0$ for moderate or even high SNR. So there must exist a value $R_{eav}^{*}$ of $R_{eff}^{\prime }\left(R_{eav}\right)=0$, where $R_{eav}^{*}$ is the value of $R_{eav}$ that maximizes the effective eavesdropping rate $R_{eff}$. So far, Lemma 1 is proved. □
